# A Screen for *rfaH* Suppressors Reveals a Key Role for a Connector Region of Termination Factor Rho

**DOI:** 10.1128/mBio.00753-17

**Published:** 2017-05-30

**Authors:** Kuang Hu, Irina Artsimovitch

**Affiliations:** Department of Microbiology and The Center for RNA Biology, The Ohio State University, Columbus, Ohio, USA; Massachusetts Institute of Technology

**Keywords:** RNA polymerase, RfaH, Rho, polarity, termination

## Abstract

RfaH activates horizontally acquired operons that encode lipopolysaccharide core components, pili, toxins, and capsules. Unlike its paralog NusG, which potentiates Rho-mediated silencing, RfaH strongly inhibits Rho. RfaH is recruited to its target operons via a network of contacts with an elongating RNA polymerase (RNAP) and a specific DNA element called *ops* to modify RNAP into a pause- and NusG-resistant state. *rfaH* null mutations confer hypersensitivity to antibiotics and detergents, altered susceptibility to bacteriophages, and defects in virulence. Here, we carried out a selection for suppressors that restore the ability of a Δ*rfaH* mutant *Escherichia coli* strain to grow in the presence of sodium dodecyl sulfate. We isolated *rho*, *rpoC*, and *hns* suppressor mutants with changes in regions previously shown to be important for their function. In addition, we identified mutants with changes in an unstructured region that connects the primary RNA-binding and helicase domains of Rho. The connector mutants display strong defects *in vivo*, consistent with their ability to compensate for the loss of RfaH, and act synergistically with bicyclomycin (BCM), which has been recently shown to inhibit Rho transformation into a translocation-competent state. We hypothesize that the flexible connector permits the reorientation of Rho domains and serves as a target for factors that control the motor function of Rho allosterically. Our results, together with the existing data, support a model in which the connector segment plays a hitherto overlooked role in the regulation of Rho-dependent termination.

## IMPORTANCE

The transcription termination factor Rho silences foreign DNA, reduces antisense transcription, mediates surveillance of mRNA quality, and maintains genome integrity by resolving transcription-replication collisions and deleterious R loops. Upon binding to RNA, Rho undergoes a rate-limiting transition from an open “lock washer” state to a closed ring capable of processive translocation on, and eventually the release of, the nascent transcript. Recent studies revealed that Rho ligands, including its cofactor NusG and inhibitor bicyclomycin, control the ring dynamics allosterically. In this work, we used a genetic selection for suppressors of RfaH, a potent inhibitor of Rho, to isolate a new class of mutations in a flexible region that connects the primary RNA-binding and ATPase/translocase domains of Rho. We propose that the connector is essential for the modulation of Rho activity by different RNA sequences and accessory proteins.

## INTRODUCTION

**FIG 1:**
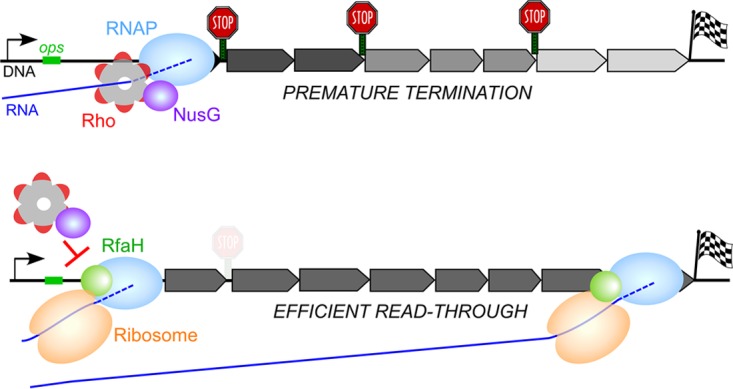
Model of activation of gene expression by RfaH. (Top) In the absence of RfaH, its target operons are poorly translated and are silenced by a joint action of Rho and NusG, which make pairwise contacts with each other. As expected for Rho-mediated polarity, the distal genes are strongly affected and the addition of BCM restores RNA levels throughout the operon (9). (Bottom) When present, RfaH is recruited at the *ops* site and remains associated with the elongating RNAP until the end of the operon. RfaH prevents NusG binding to the transcription complex and is thought to directly load the ribosome onto RNA. A strong antipolar effect of RfaH is comparable to that of BCM (9).

*Escherichia coli* RfaH is a sequence-specific paralog of the housekeeping transcription elongation factor NusG. Unlike NusG, which is present in all domains of life (1), RfaH orthologs are restricted to *Bacteria*, where they activate the expression of cell wall components, pili, toxins, capsules, and antibiotic biosynthesis clusters in diverse phyla, including *Enterobacteriaceae*, *Bacteroidetes*, and *Firmicutes* (1). Like all other NusG-like proteins, RfaH and NusG bind to the β' subunit clamp helix motif to increase RNA polymerase (RNAP) processivity but have opposite effects on gene expression (Fig. 1). *E. coli* NusG silences the expression of foreign DNA by potentiating Rho-dependent termination (2); this is an essential function of NusG (3) mediated by direct contacts with the termination factor Rho (4). In contrast, *E. coli* RfaH increases the expression of several horizontally acquired operons that contain an *ops* sequence in their leader regions; *rfaH* null mutants are viable but have defects in virulence, hypersensitivity to antibiotics and detergents, and altered susceptibility to bacteriophages (1).

RfaH is recruited to RNAP paused at the *ops* site and remains bound to the transcription elongation complex until its dissociation at an intrinsic terminator (5). RNAP-bound RfaH reduces the efficiency of Rho-dependent termination by three distinct mechanisms. First, RfaH decreases RNAP pausing, thereby inhibiting Rho kinetically (6). Second, RfaH excludes NusG from binding to RNAP (5) and is thus expected to reduce termination at a subset of sites that are potentiated by NusG (2). Third, RfaH is thought to recruit the ribosome to mRNAs and subsequently couple transcription to translation via direct interactions with ribosomal protein S10 (7); the coupled ribosome will shield the nascent mRNA from Rho (8). Together, these activities essentially abrogate Rho-mediated polarity; e.g., the expression of the *rfb* operon, which is silenced by Rho, increases ~300-fold in the presence of RfaH (9).

While the molecular mechanism of RfaH has been extensively studied, its cellular context is poorly understood. An early report by Farewell et al. (10) identified 10 *rfaH* suppressors in *Salmonella* by selecting for resistance to bacteriophage Ffm. Consistent with an expectation that reduced Rho-dependent termination may be able to compensate for the lack of RfaH, three of these suppressors were in *rho* and one mapped near the *rpoBC* (and *nusG*) genes, but their identities were not determined. The remaining suppressors could not be mapped by linkage to *rfaH* and the *rfa* operon, a known RfaH target.

In this work, we carried out a selection for suppressors that restored the ability of Δ*rfaH* mutant *E. coli* strain MG1655 to grow in the presence of sodium dodecyl sulfate (SDS). This selection could identify several classes of suppressors that (i) reduce Rho-dependent termination, (ii) enable RfaH-independent ribosome recruitment, (iii) reduce the intracellular concentration of SDS, and (iv) increase the transcription of RfaH-dependent operons, e.g., by activating their promoter. The first two classes of suppressors would provide mechanistic insights into the regulation of gene expression by RfaH.

We were particularly interested in identifying unknown players involved in RfaH control. We also wondered whether termination at many cryptic sites unmasked in the absence of translation imposes different requirements for Rho, compared to a single, strong terminator commonly used to isolate termination-altering mutations (11). Analysis of spontaneous SDS-resistant *rfaH* suppressors identified changes in functionally important regions of Rho, the β' subunit of RNAP (*rpoC*), and the nucleoid-associated protein H-NS (*hns*). Consistent with our expectations, we also isolated mutations in a nonconserved region of *rho* that was not highlighted by previous genetic analyses. We propose that these mutations interfere with the allosteric control of Rho by RNA signals and regulatory factors.

## RESULTS

### Selection strategy.

The loss of RfaH results in lipopolysaccharide chains of various lengths and confers sensitivity to bile salts, detergents, and antibiotics (12). A polar mini-Tn*5* insertion into MG1655 *waaQ* (*rfaQ*), the first gene in the *waa* operon regulated by RfaH (5), produces similar phenotypes (13). Consistent with these reports, we found that the Δ*rfaH* mutant MG1655 strain was more sensitive to bile salts, novobiocin, and nalidixic acid than the wild-type (WT) isogenic strain. However, growth on SDS plates showed the greatest differential; while MG1655 grew on 10% SDS (the highest concentration tested), its Δ*rfaH* mutant derivative failed to grow on 0.016% SDS (the lowest concentration tested). Therefore, we selected for spontaneous suppressors by plating three independent overnight cultures of the Δ*rfaH* mutant strain on LB plates supplemented with 0.5% SDS at 37°C. SDS-resistant mutants arose at an apparent frequency of 1.80E-07, some of which could be clonal variants. To increase our chances of recovering mutants with different properties, we carried out phenotypic screens of ~300 mutants to identify colonies with different morphologies (small/large and round/flat) and sensitivities to antibiotics (see Fig. S1 in the supplemental material). A subset of suppressor alleles was sensitive to novobiocin, an inhibitor of DNA gyrase subunit B; early studies linked *rho* defects to novobiocin sensitivity (14). We chose 32 suppressors that appeared phenotypically distinct for further analysis.

10.1128/mBio.00753-17.1 FIG S1  Initial screening of *rfaH* suppressor variants for sensitivity to novobiocin. The spontaneous suppressors from one plate were patched onto plates supplemented with 0.25% SDS or 60 µg/ml novobiocin. All but one of the novobiocin-sensitive variants were subsequently mapped to *rho*. Download FIG S1, PDF file, 0.3 MB. Copyright © 2017 Hu and Artsimovitch. 2017 Hu and Artsimovitch This content is distributed under the terms of the Creative Commons Attribution 4.0 International license.

### Identification of suppressors in the transcription apparatus.

RfaH acts by inhibiting Rho (9), and mutations that compromise Rho-dependent termination would be expected to compensate for the lack of RfaH. Since the discovery of Rho in 1969, numerous screens for Rho mutants have been performed, identifying the key functional regions (15). In addition to mutations in *rho*, mutations in *rpoBC*, *nusG*, and *nusA* have been shown to affect Rho function (16, 17). We thus tested whether any of the suppressors identified mapped to these genes by P1 transduction from the Keio collection strains, in which a kanamycin resistance marker replaced a nearby locus (18). We determined linkage between each selected Keio marker and suppressor alleles by calculating the frequency of SDS-sensitive, Kan-resistant colonies following transduction into the original SDS-resistant suppressor strain. Using this approach, we were able to map one mutation to *rpoC* and 13 mutations to *rho* (10 unique; see Table S1) by linkage to *thiH*::Kan and *wzzE*::Kan, respectively. None of the mutations mapped to *nusA* (*argG*::Kan), *nusG* (*thiH*::Kan), or the *waa* operon (*waaO*::Kan).

10.1128/mBio.00753-17.7 TABLE S1  SDS sensitivity of Δ*rfaH* suppressors. (Superscript 1) The position of a mutation is indicated relative to the +1 position of the open reading frame. In the case of *hns*, +1 corresponds to the N-terminal Met residue that is absent from the mature protein. (Superscript 2) To assess resistance to SDS, overnight cultures were diluted at 10^−2^, 10^−3^, 10^−4^, 10^−5^, and 10^−6^ with LB. Each diluted culture was spotted onto LB plates containing 0.003% (1), 0.016% (2), 0.08% (3), 0.4% (4), or 2% (5) SDS. The score indicates growth without apparent inhibition at the highest SDS concentration; e.g., 1+ indicates efficient growth at 0.003% and 5+ indicates efficient growth at 2% SDS. Download TABLE S1, DOCX file, 0.02 MB. Copyright © 2017 Hu and Artsimovitch. 2017 Hu and Artsimovitch This content is distributed under the terms of the Creative Commons Attribution 4.0 International license.

#### RpoC.

The single suppressor in *rpoC* was a two-nucleotide insertion at codon 1361 of the β' subunit of RNAP. This insertion results in a frameshift in which the C-terminal 46 β' residues are replaced with a heterologous 23-residue sequence (PVTRTTRIVCVAVLRVKLRLHRR). This extension is identical to the one present in RpoC397, in which the frameshift is a result of the deletion that removes 52 C-terminal β' residues (19). We are currently characterizing the phenotypes of the Δ46 enzyme *in vivo* and *in vitro*.

#### Rho.

**FIG 2:**
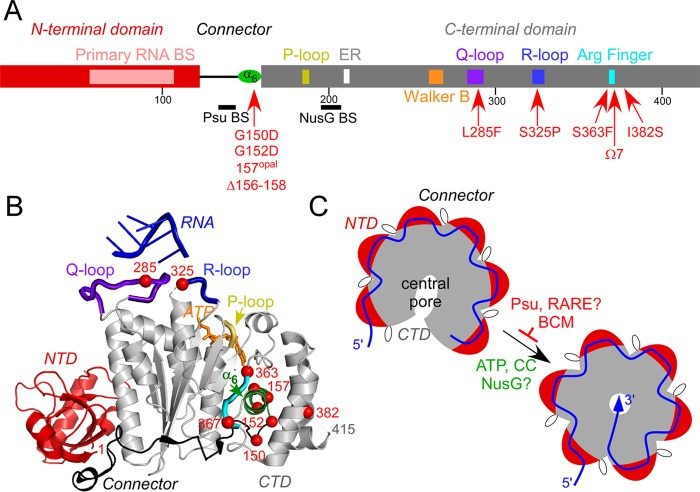
Structure and rearrangements of Rho. (A) Schematic diagram of Rho with the domain boundaries and key regions (15) indicated. The NTD, CTD, and connector are red, gray, and black, respectively. The key Rho elements are shown as colored boxes. The suppressor mutants isolated in this work are shown below the sequence. BS, binding site; ER, a motif of two residues, Glu and Arg. (B) Structure of the Rho monomer (PDB code 3ICE). The NTD, CTD, and key CTD regions are colored as in panel A; the bound ATP (orange) and RNA in the central pore (blue) are also shown. The Cα atoms of the mutated residues are shown as red spheres. (C) The apo Rho hexamer exists in an open state in solution and undergoes a rigid-body rearrangement into the closed state in the presence of RNA and ATP (27), trapping the RNA in the central channel. The CC dinucleotide contacts with the NTD favor the transition, whereas BCM inhibits it (27). Other Rho ligands could affect the conformational change (as shown by a question mark).

We identified two insertions, a deletion, and point mutations in the *rho* locus. An IS*2* insertion in *rhoL* would be expected to reduce the levels of Rho. Four mutations in the coding region are located in highly conserved motifs previously implicated in Rho function (Fig. 2). L285F is in the Q loop, and S325P is in the R loop, the two loops that face the central pore of Rho and comprise its secondary RNA-binding site. S363F and a seven-residue (EELLTTQ) insertion after residue 367 are in the Arg finger (20). The latter variant conferred very weak resistance to SDS (see Table S1) and was excluded from the subsequent analysis. The I382S substitution is in a less conserved region, but a similar I382N substitution dramatically increased readthrough *in vivo* but not *in vitro* (11). In addition to these “expected” mutations, we isolated four mutations (two of them more than once) in a nonconserved region of Rho that connects the N-terminal and C-terminal domains (NTD and CTD, respectively) of Rho. These changes are immediately upstream and within the α_6_ helix in the CTD (residues 153 to 166) that is adjacent to the connector. Mutations in this region have not been reported previously; we hypothesize that these changes alter domain rearrangements required for Rho function (see Discussion). All *rho* alleles conferred increased sensitivity to novobiocin (see Fig. S2), consistent with a previous report (14).

10.1128/mBio.00753-17.2 FIG S2  Novobiocin sensitivity of *rfaH*^sup^ variants. Antibiotic disc diffusion assays were performed with the Δ*rfaH rho*^+^ *hns*^+^ strain (WT) and selected alleles of *rho* and *hns*. The diameters (in millimeters) of zones with no growth (clear) are shown as black bars; with *rho* S363F, an area of weak inhibition (hazy appearance, indicated by a gray bar) was observed. No visible zone of inhibition is shown as a value of <7 mm; the diameter of the disc is 6 mm. Each assay was performed at least three times; the errors bars are the standard deviations. Download FIG S2, PDF file, 0.1 MB. Copyright © 2017 Hu and Artsimovitch. 2017 Hu and Artsimovitch This content is distributed under the terms of the Creative Commons Attribution 4.0 International license.

### Most SDS-resistant mutations are in the *hns* gene.

To map the remaining mutants, we used random Tn*5*::Tet libraries (21). Using this approach, we mapped 16 mutations to *hns* (7 unique) and 1 to *yciC* (see Table S1). The remaining suppressor could not be mapped with different Tn*5* libraries, suggesting that it contained more than one mutation required to confer the SDS resistance phenotype. The *yciC* gene encodes a predicted inner membrane protein. It is unclear how a missense mutation (A83T) in *yciC* restores SDS resistance in the *rfaH* background, as the function of YciC is unknown.

Mutations in *hns* could affect the expression of RfaH-dependent genes in several ways. First, H-NS silences the transcription of the *waa* operon (22) and loss-of-function *hns* mutants could compensate for the lack of RfaH by increasing the amount of *waa* RNA. Second, H-NS may cooperate with Rho to inhibit the expression of some genes (23). Changes at the dimer interface, which encompasses residues 2 to 47 and 58 to 84, will be expected to alter the structure of H-NS filaments, in turn affecting Rho function. Surprisingly, although L26P and E74K H-NS variants have been shown to restore viability to strains carrying defective *rho* and *nusG* alleles (24) and are thus expected to increase Rho-dependent termination, we isolated substitutions at identical (L26P) and adjacent (L75Q) positions that likely reduce Rho-dependent polarity in the *waa* operon. Furthermore, the *hns* suppressor alleles were different in the extent of SDS resistance (see Table S1) and sensitivity to novobiocin (see Fig. S2), implying that their underlying mechanisms are distinct. Elucidation of the mechanism of suppression will necessitate an in-depth analysis of the effects of *hns* and other nucleoid-associated proteins on the regulation of RfaH-controlled genes, which we intend to undertake in the future. In this work, we focused on novel Rho variants identified by our suppression screen.

### Substitutions in Rho reduce transcription termination and confer growth defects.

**FIG 3:**
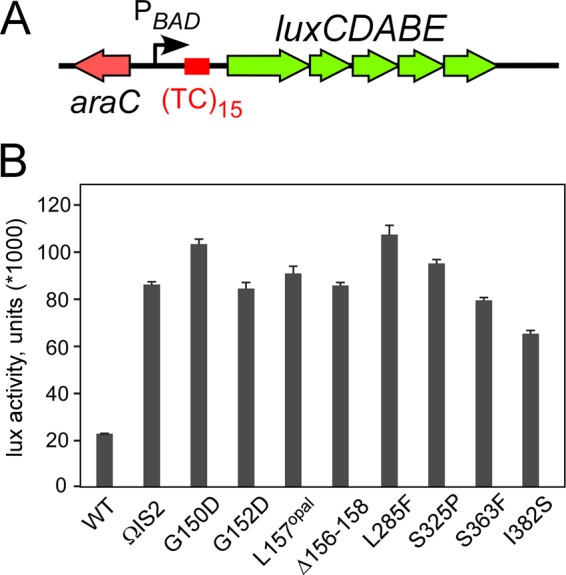
Termination defects of Rho variants *in vivo*. (A) A reporter containing a synthetic TC_15_ *rut* element between the arabinose-inducible P_BAD_ promoter and the *lux* operon. (B) Analysis of the effects of the original suppressor *rho* mutations (strains 303 to 344) on *lux* operon expression. The results are expressed as luminescence corrected for the cell densities of individual cultures, in thousands. The data represent the average of three independent experiments ± the standard deviation.

It is logical to assume that the *rho* suppressors isolated compensate for the lack of RfaH by reducing Rho-dependent termination. To test this assumption, we compared the effects of *rho* alleles on reporter expression *in vivo*. Defects in Rho function lead to the overreplication of most ColE1 plasmids, which replicate via an R-loop intermediate, whereas pSC101 plasmids are stably maintained (24). We constructed a pSC101-based vector in which a *lux* operon from *Photorhabdus luminescens* was cloned downstream from the arabinose-inducible P_BAD_ promoter and a synthetic (TC)_15_ terminator (Fig. 3A) that functions efficiently *in vivo* and *in vitro* (25). Following transformation into selected suppressor strains, we measured the luciferase activity in exponentially growing live cells. We observed that all mutations in *rho* led to greater *lux* expression than that in the *rho*^+^ Δ*rfaH* strain (Fig. 3B). Since Rho is essential in *E. coli*, we expected that the termination-deficient *rho* mutants would exhibit growth defects. Consistently, all *rho* mutations reduced growth (see Fig. S3). The I382S and L285F alleles were the least and most defective, respectively, an observation consistent with their relative effects on termination; however, there was no correlation between the growth and termination defects of all mutants (Fig. 3B).

10.1128/mBio.00753-17.3 FIG S3  Rho mutants exhibit growth defects *in vivo*. Single colonies were inoculated into 3 ml of LB and incubated at 37°C with aeration. After 8 h of growth, 4 μl of each culture was mixed with 196 μl of LB in a 96-well plate (CellStar; Greiner). The plates were sealed with gas-permeable moisture barrier seals (4titude) and incubated in an iEMS Reader MF (Labsystems) at 37°C and 300 rpm. The OD_600_ was measured every 30 min. Results were analyzed with Microsoft Excel. Each strain was grown in triplicate, and average values are shown. Download FIG S3, PDF file, 0.05 MB. Copyright © 2017 Hu and Artsimovitch. 2017 Hu and Artsimovitch This content is distributed under the terms of the Creative Commons Attribution 4.0 International license.

### Suppressors do not dramatically decrease Rho levels.

Substitutions in Rho could affect its function or reduce its levels/stability. An IS*2* insertion in the *rho* leader would be expected to reduce *rho* expression, whereas substitutions in the conserved regions could interfere with Rho binding to RNA or inhibit ATP hydrolysis/translocation. To determine whether the defects observed are due to reduced Rho levels, we performed Western blotting with anti-Rho polyclonal antibodies. We found that none of the alleles changed Rho levels more than 2.5-fold relative to those of the WT; these small effects are likely explained by autoregulation of Rho expression (26). IS*2* insertion and L157^opal^ substitutions decreased Rho levels to 40%, whereas Δ156-158 and S363F had smaller effects (see Fig. S4). In contrast, other substitutions increased Rho levels. Interestingly, we observed very efficient ribosome readthrough of the stop codon in the L157^opal^ mutant (see Fig. S4). Our initial attempts to identify the substituted residue were not successful, and since the reduced Rho level would be sufficient to explain the termination-altering phenotype of this mutant, we did not pursue this line of analysis. We conclude that the termination defects of most Rho mutants are conferred by the substituted residues.

10.1128/mBio.00753-17.4 FIG S4  Immunoblot analysis of levels of variant Rho proteins in cultures grown overnight in LB. Samples were normalized for cell density and subjected to electrophoresis through 4 to 12% Novex bis-Tris denaturing polyacrylamide gel. Proteins were transferred to nitrocellulose membranes (Roche Diagnostics), which were probed with anti-Rho (1:10,000 dilution) antiserum, followed by horseradish peroxidase-conjugated anti-rabbit antibodies (1:10,000 dilution; GE Health). A signal was developed with Clarity Western ECL substrate (Bio-Rad) and imaged with a ChemiDoc XRS+ System and Image Lab 5.2.1 software (Bio-Rad). The position of the Rho protein was identified with the purified sample (not shown); weakly cross-reacting bands shown by arrows are indicative of loading consistency and were used for normalization. The signal was quantified by ImageJ, and the level of WT Rho was set at 1. The assay was repeated two to four times, giving reproducible results, and a subset of variants analyzed on a different gel (separated by a black line) is shown. Download FIG S4, PDF file, 1 MB. Copyright © 2017 Hu and Artsimovitch. 2017 Hu and Artsimovitch This content is distributed under the terms of the Creative Commons Attribution 4.0 International license.

### Substitutions in the connector lead to defects at a natural terminator.

**FIG 4:**
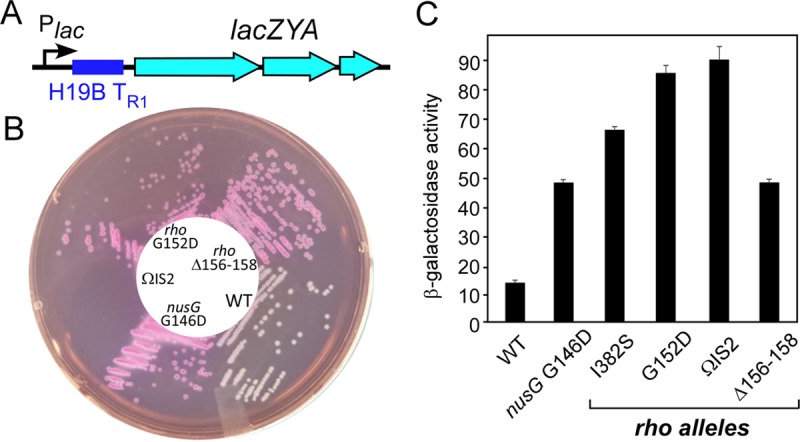
Transcription through a phage H19B T_R1_ Rho-dependent terminator. (A) A chromosomal reporter with the phage H19B terminator inserted into the leader region of the *lac* operon. (B) A plate assay on MacConkey lactose agar. The selected *rho* alleles were moved into the test strain by P1 cotransduction with the *ilvC*::Kan marker, resulting in strains 404 to 418. (C) Liquid β-galactosidase assay. The data represent the average of three independent experiments ± the standard deviation.

The (TC)_15_ element is a near-perfect *rut* (Rho utilization) element, whereas Rho is able to act on very diverse sequences *in vivo*, and strong Rho-dependent sites have not been identified in RfaH-controlled operons. To confirm that the isolated suppressors are defective in termination at a natural site, we used a chromosomal β-galactosidase reporter previously used to identify defective *rho* alleles (11). In this strain, a λRS45 lysogen carries a T_R1_ terminator from lambdoid phage H19B between the P_*lac*_ promoter and the *lacZYA* operon (Fig. 4A). We moved *rho* mutations linked to the *ilvC*::Kan marker into the test strain (*rfaH*^+^). The suppressor mutations in *rho* formed red colonies on MacConkey lactose agar (Fig. 4B), similarly to *nusG* G146D, which reduces the efficiency of Rho-dependent termination (24). β-Galactosidase activities measured in exponentially growing cells confirmed these observations (Fig. 4C).

These results show that the α_6_ and adjacent residues play an important role in the cellular function of Rho, and their substitution confers defects at both synthetic and natural terminators. These defects are comparable to that caused by the substitution of Ile382, a residue that was shown to be important for Rho-dependent termination (11). However, we were unable to transduce L285F, S325P, and S363F alleles into the test strain, suggesting that the original suppressor strains carry second-site mutations that allow the growth of these presumably strongly deleterious *rho* variants that contain substitutions in the invariant (15) residues of the Q loop, R loop, and Arg finger. Consistent with this interpretation, we could not transduce L285F, S325P, and S363F alleles (linked to *ilvC* or *wzzE*) into the original Δ*rfaH* mutant strain (IA228), whereas other alleles were moved easily, conferring resistance to SDS and exhibiting termination defects (see Fig. S5).

10.1128/mBio.00753-17.5 FIG S5  Analysis of the effects of the suppressor *rho* mutations following P1 transduction into Δ*rfaH* mutant MG1655 strains 431 to 440 on *lux* operon expression *in vivo*. The assay was performed as described in the legend to Fig. 3. The results are expressed as luminescence corrected for the cell densities of individual cultures. The data represent the average of three independent experiments ± the standard deviation. Download FIG S5, PDF file, 0.04 MB. Copyright © 2017 Hu and Artsimovitch. 2017 Hu and Artsimovitch This content is distributed under the terms of the Creative Commons Attribution 4.0 International license.

### The *nusG* G146D allele suppresses *rfaH.*

RfaH excludes NusG from RNAP transcribing *ops*-containing operons (5). In the absence of RfaH, NusG would be expected to take the place of RfaH and increase Rho-dependent termination (8). In this scenario, decreasing Rho-NusG interactions should compensate for the loss of RfaH. To test this prediction, we used a *nusG* G146D allele that has been shown to reduce Rho-dependent termination (24). When transduced into a Δ*rfaH* mutant strain, the *nusG* G146D allele restored growth on 0.5% SDS (see Fig. S6). This result confirms that RfaH acts in part by directly competing with NusG. The loss of RfaH would unmask numerous potential Rho loading sites in the *waa* operon (and others) whose utilization may be dependent on NusG (2).

10.1128/mBio.00753-17.6 FIG S6  A NusG G146D variant is an efficient suppressor of the Δ*rfaH* SDS-sensitive phenotype. The Δ*rfaH* mutant MG1655 strain was designated the WT, and all of the alleles indicated were constructed in this strain background. The *rfaH*^+^ MG1655 strain is shown for comparison. LB plates supplemented with 0.5% SDS were incubated overnight at 37°C. Download FIG S6, PDF file, 1 MB. Copyright © 2017 Hu and Artsimovitch. 2017 Hu and Artsimovitch This content is distributed under the terms of the Creative Commons Attribution 4.0 International license.

## DISCUSSION

In this work, we screened for suppressors of the *E. coli rfaH* deletion phenotypes. On the basis of previous results with *Salmonella* (10) and the primary mode of RfaH action as an antagonist of Rho (9), we expected to identify suppressors in *rho*, *rpoBC*, and perhaps *nusG* that alleviate Rho-mediated polarity in *waa*, in which a polar mini-Tn*5* insertion phenocopies the effects of *rfaH* (13). Loss-of-function mutations in *hns* could also compensate for the lack of RfaH because H-NS represses the transcription initiation of (22), and is enriched in (2), the *waa* operon, where it could act synergistically with Rho. Consistently, our analysis identified substitutions in key Rho and H-NS regions that could lead to a partial or total loss of function. However, we also identified changes in *rho*, *rpo*, and *hns* regions not expected to lead to defects in Rho-dependent termination (see above). In this study, we focused on novel mutations in the connector region of Rho, a nonconserved unstructured linker between the NTD and CTD of Rho. We hypothesize that these substitutions interfere with communication between the Rho domains, underscoring the importance of the allosteric control of Rho function illustrated by several recent reports (27–29).

### Structural rearrangements of the connector.

Rho is a hexameric RecA family translocase composed of two domains connected by a flexible ~30-residue linker (Fig. 2B). The NTD harbors a primary RNA-binding site, and the CTD contains the secondary RNA-binding site and ATPase and helicase determinants. Rho-dependent termination is a highly tunable event that can be separated into four sequential steps (reviewed in reference 8). First, residues in the primary RNA-binding site interact with pyrimidine dinucleotides in a *rut* element; the *rut* composition is a key determinant of Rho termination, with Rho affinity ranging widely, depending on the *rut* sequence (8). Second, the growing RNA downstream from the bound *rut* site is threaded into the central channel, where the secondary RNA-binding site residues from the Q and R loops make direct contacts with RNA. Binding of RNA to the central channel triggers, in the presence of ATP, a conformational change from an open to a closed, translocation-competent state in which the RNA is trapped inside the hexamer (Fig. 2C). Third, the closed hexamer engages in stepwise ATP-powered translocation 5′ to 3′ along the RNA toward the RNAP while maintaining the contacts with the *rut* element in a tethered-tracking mode. Finally, upon reaching the paused RNAP, Rho extracts the nascent transcript to trigger the TEC dissociation. Following the RNA release from the RNAP, the Rho ring must open to reset the cycle.

The transition from the open to the closed state is thought to represent the rate-limiting step and is thus expected to be a key target for regulation. Recent studies showed that Rho exists in the open state in solution even in the presence of physiological levels of ATP and that the ring closes only upon the binding of RNA to the secondary sites in the CTD (27). While U_12_ RNA bound in the central pore was sufficient to induce the transition, occupancy of the primary sites in the NTD promoted ring closure around a suboptimal A_12_ RNA, implying allosteric communication between the two domains. Accordingly, comparison of the open and closed Rho structures reveals that the two domains and the connector may undergo significant rearrangements during ring closure. The N and C termini become more flexible, while the two “handles” of the connector, residues 126 to 129 and 147 to 152, become ordered in the closed state. The α_6_ helix (residues 153 to 166) located at the end of the connector is rotated 40° in a closed Rho structure obtained with a long 30-mer RNA (30) but not in those with shorter RNAs (27, 31), suggesting that different ligands could promote different structural transitions of the ring. The observed changes in the connector are likely essential for the attainment of the translocation-competent Rho conformation. Substitutions or ligands that restrict these movements would be expected to inhibit Rho-dependent termination.

We identified four defective Rho variants with changes in and immediately upstream of α_6_, which is sandwiched between α_7_ and α_16_. This region (residues 150 to 158) is poorly conserved among Rho homologs (20) but is enriched (with a loose consensus, GNGSTEDLT) in residues that are favored in natural and engineered protein linkers (32). Glycine is strongly preferred in flexible regions, but charged residues are also tolerated. Gly150 and Gly152 are located in the region disordered in the open hexamer structure (33), and their substitutions for Asp would be expected to rigidify the α_6_ junction. Changes in α_6_, such as a Leu157X substitution and the deletion of three residues (156 to 158), could lead to repositioning of the P loop located at the end of α_7_. Mori et al. isolated a defective D156N substitution that did not compromise Rho binding to RNA, suggesting a postbinding defect (40). Substitution of the first α_6_ residue, S153Y, which was isolated in combination with a P103L substitution, could not be engineered alone (41). We hypothesize that the S153Y substitution restricts the mobility of the connector-α_6_ junction, acting similarly to G150D and G152D.

### Tuning Rho-dependent termination.

In contrast to intrinsic termination, which depends primarily on a signal in the nascent RNA, Rho-dependent termination is only loosely dependent on the RNA sequence. Rho preferentially interacts with short, C-rich sequences in both the primary and secondary sites (28), but Rho affinities for C-rich sequences vary greatly and Rho can terminate transcription at sites with lower C content (2). Broad sequence specificity is likely a prerequisite for many diverse roles that Rho has been shown to play. In addition to terminating the transcription of some structural genes, Rho terminates the transcription of poorly translated RNAs, such as horizontally transferred foreign genes, antisense RNAs, or mRNAs bearing early stop codons, resolves R loops, and ensures genome stability by reducing replication-transcription collisions (see reference 8 and references therein). Thus, Rho has to act at emerging problem sites in addition to genetically programed sites, and its recruitment to the nascent RNA is strongly context dependent.

Productive recruitment of Rho requires an extended >70-nucleotide-long segment of RNA that is devoid of strong secondary structures and RNA-bound proteins, followed by ring closure. Termination by Rho can be tuned by nascent RNA sequences and *trans*-acting RNA and protein cofactors that can act directly or indirectly (8, 34, 35). *E. coli* NusG is the best-characterized activator of Rho. Among more than 1,000 Rho-dependent sites in MG1655, those with poor *rut* elements (that possess low C>G ratios) require NusG for efficient termination (2), implying that NusG binding to the Rho CTD may mimic an effect of an optimal C-rich signal or ligand binding to the primary sites (27). H-NS, which colocalizes to many Rho release sites, also potentiates Rho-mediated RNA release (2). Diverse regulators that inhibit Rho have been characterized (8, 34, 35). Some antiterminators are recruited only to their target operons to modify the RNAP into a termination-resistant state (36). Others, such as Psu and Hfq (37, 38), appear to directly inhibit Rho via protein-protein interactions. While their detailed mechanisms remain to be determined, some of these regulators may utilize several independent modes of inhibition to ensure potent activation of their target genes.

### Rho modulators may act allosterically.

Recent structural and biochemical studies suggest that many, if not most, Rho ligands act allosterically. BCM, which has been argued to act as a noncompetitive ATP-binding inhibitor of Rho, has recently been shown to block ring closure (27). Conversely, NusG (29) and RNA bound to the primary sites (27) appear to promote ring closure. Whereas the sensitivity to substitutions signifies the mechanistic role of the connector, mounting evidence also points to the key role of this region in Rho regulation. Psu, a phage-encoded Rho antagonist, binds to the region encompassing residues 139 to 153 (38). The Psu dimer has been proposed to bridge two Rho protomers to sterically block the central RNA-binding channel and thereby translocation (38) but may also restrict connector movement and thus inhibit the closed-to-open transition. Interestingly, a P167L substitution at the C terminus of α_6_ restored the function of defective Psu (38). NusG potentiates Rho-mediated termination at suboptimal *rut* sites (2). Several underlying mechanisms have been proposed, but the existence of substitutions at the opposite ends of the connector that mimic (29, 39) or abolish (33) the effect of NusG suggests that NusG may stimulate Rho activity by realigning the domains to facilitate ring closure. We speculate that a recently discovered RARE element that likely binds to the NTD (34) and Hfq (37) may antagonize Rho activity by inhibiting ring closure.

These data support a model in which the connector segment plays a hitherto unknown regulatory role. Structural evidence shows that the connector is a mobile tether, and our present findings suggest that it may be what enables facile transitions between the open and closed states. The connector is present in diverse Rho homologs, across which the majority of its sequence is not conserved (20). If our conjecture regarding the functional importance of the connector is correct, the variability of its sequence would further suggest that it may be a target for species-specific regulators that modulate Rho activity. Such modulation could be used to respond to unique cellular contexts that may differ among different bacteria, particularly those in which Rho is dispensable. Our isolation of mutations in the variable region of the connector might justify extending this study to other bacterial species to explore the possibility of such specificity.

### Changes in the connector act synergistically with BCM.

We propose that connector flexibility is essential for Rho ring closure; we hypothesize that Gly-to-Asp substitutions at positions 150 and 152 reduce this flexibility but still permit the domain rearrangements, whereas more “drastic” changes will likely be lethal, e.g., S153Y in the absence of compensatory changes in the primary RNA-binding region (41).

**FIG 5:**
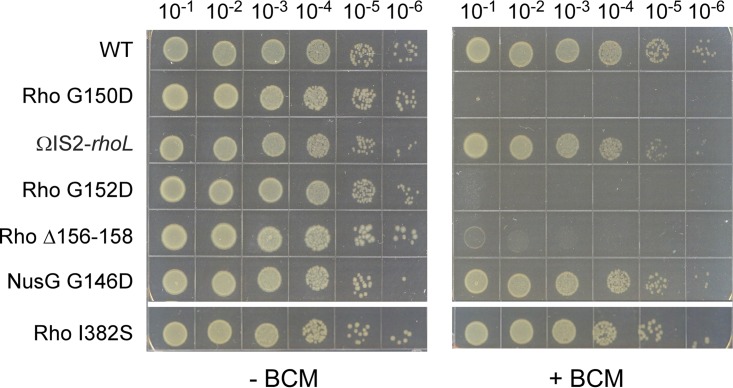
Connector mutants are hypersensitive to BCM. Serial 10-fold dilutions of exponentially growing cultures of a Δ*rfaH* mutant strain carrying the mutations indicated (strains 431 to 440) were plated on LB (left) or LB supplemented with 10 µg/ml BCM (right) and incubated overnight at 37°C. A set representative of three independent experiments is shown.

If this were true, the G150D and G152D substitutions would be expected to act similarly to BCM, inhibiting the transformation into the active, translocation-competent state and thereby conferring increased sensitivity to BCM *in vivo*. Indeed, we found that the G150D and G152D alleles and, to a lesser extent, the Δ156-158 *rho* allele caused severely impaired growth at low (10 µg/ml) BCM concentrations that fully supported the growth of the WT strain (Fig. 5). In contrast, reducing the level of WT Rho (ΩIS*2*), inhibiting Rho interactions with NusG (G146D substitution), or inhibiting Rho function by substitution of Ile382 did not lead to BCM hypersensitivity (Fig. 5). These observations are consistent with our hypothesis that BCM and the connector affect the same step in the Rho mechanism.

### Why did we isolate connector mutants?

Most screens for defective *rho* mutants have been carried out with strong terminators placed in front of a reporter gene. These screens identified mutations in several key regions of Rho (Fig. 2A). In our selection, mutations in these regions were also recovered, along with a new class of mutations in the connector (4 out of 10). We note that a screen for suppressors of polarity in *Salmonella* also identified a connector mutation (39). Although our selection was by no means saturating, the repeated recovery of two of these mutants argues against a serendipitous explanation and implies that a different basis for our selection could explain this bias. We assume that compromised *waa* operon expression is the underlying reason for the SDS sensitivity of the *rfaH* mutant strain (see above). In WT MG1655, *waa* is a poor target for Rho; it has a low frequency of C residues (17.4%) and is devoid of NusG that is excluded by RfaH. The loss of RfaH would be expected to permit NusG binding to RNAP and abolish ribosome recruitment, potentiating Rho-depending termination. Because of the absence of demonstrably strong Rho termination sites (2), we propose that, rather than using its most potent mode of gene silencing (at a single, dominant early site), Rho silences *waa* expression by inducing termination at many weak sites along the operon. The absence of a strong RNA-binding site should increase the dependence of regulation on the allosteric communication between the two domains and therefore the functionality of the linker.

## MATERIALS AND METHODS

### Strains.

For the strains used in this study, see Table S2. Unless indicated otherwise, cells were grown in LB or EZ Rich Defined Medium (Teknova) with 0.2% glycerol (EZRDM-G) at 37°C. For plates and top agar, LB was supplemented with 1.5 and 0.75% (wt/vol) agar, respectively. When necessary, spectinomycin (20 μg/ml), BCM (10 μg/ml), kanamycin (30 μg/ml), tetracycline (20 μg/ml), or SDS (0.003 to 2%) was added to the growth medium.

10.1128/mBio.00753-17.8 TABLE S2  Strains used in this work. (Superscript 1) IA228 was constructed from IA227 by P1 transduction of the *rfaH*::Kan allele from the Keio collection and subsequent flipping out of the kanamycin resistance cassette. (Superscript 2) Strains IA303 to IA352 are original isolates of spontaneous SDS-resistant (SDS^r^) suppressors of IA228. (Superscript 3) Strains 383 and 384 were constructed by P1 transduction of *rpoC*::Kan closely linked to the *nusG* G146D allele from IA381 into IA227 and IA228. The transductants were sequenced to confirm the presence of the G146D substitution. (Superscript 4) Strains IA404 to IA418 were constructed by P1 transduction of the *ilvC*::Kan marker from IA228 and its SDS^r^ derivatives into JG5147 (IA392). The *lac*^+^ transductants were sequenced to confirm the presence of *rho* mutations. (Superscript 5) Strains IA431 to IA440 were constructed by P1 transduction of the *ilvC*::Kan marker from IA228 and its SDS^r^ derivatives into IA228 (SDS^s^). The SDS^r^ transductants were sequenced to confirm the presence of *rho* mutations. Download TABLE S2, DOCX file, 0.02 MB. Copyright © 2017 Hu and Artsimovitch. 2017 Hu and Artsimovitch This content is distributed under the terms of the Creative Commons Attribution 4.0 International license.

### Plating efficiency assays.

Single colonies were inoculated into 3 ml of LB medium and incubated at 37°C with aeration overnight. The cultures were diluted at 10^−2^, 10^−3^, 10^−4^, 10^−5^, and 10^−6^ with LB. Five microliters of each diluted culture was spotted onto LB plates containing 2, 0.4, 0.08, 0.016, or 0.003% SDS.

### Mapping of mutant alleles.

To map the *rfaH* suppressors, linkage to selected markers (linked to the Kan resistance gene in the Keio collection [18]) was tested by P1vir transduction. Suppressors in *rho* (linked to *ilvC*::Kan and *wzzE*::Kan) and *rpoC* (*thiH*::Kan) were identified by this approach. To map the remaining suppressors, we used random Tn*5*::Tet libraries (a gift from Natacha Ruiz). A P1 lysate from a pool of mutants carrying randomly inserted mini-Tn*tet* cassettes in the chromosome of WT strain MC4100 was used as a donor of WT alleles in P1 transductions where the recipients were Δ*rfaH* mutant strains carrying the SDS-resistant suppressors. We then screened for transductants that lost resistance to SDS. PCR of the chromosomal DNA was performed with an arbitrary primer (5′-GGCCACGCGTCGACTAGTACNNNNNNNNNNACGGC) and a transposon-specific primer (5′-CCTTCATGTTAACCCCTCAAGCTCAGGGG), and the resulting PCR products were sequenced to identify the mutated region. P1vir transduction was then used to finely map the suppressors. To identify the mutations, PCR amplification of the affected region with locus-specific primers and sequencing were used. To confirm that these *rfaH*^sup^ mutations conferred the suppressor phenotype, cotransduction was used to move the nearby marker and the mutant alleles into IA228.

### Disc diffusion assays.

Disc diffusion assays were performed by pouring a mixture of 100 μl of an overnight culture and 4 ml of LB top agar over LB agar plates. After the top agar solidified, 6.5-mm antibiotic disks (BD BBL Sensi-Disk Susceptibility Test Disks) containing novobiocin (30 μg) were placed on top. After overnight incubation at 37°C, zones of clearance around the disks were measured. The data shown are representative of at least three independent experiments.

### Luciferase reporter assays.

Strains defective in Rho-dependent termination are susceptible to killing because of the overreplication of many common plasmids; in contrast, pSC101-based vectors can be stably maintained in these strains (24). We therefore constructed reporter plasmid pHK2, in which the pSC101 origin of replication and spectinomycin resistance gene were combined with the *araC*-P_BAD_TC_15_*luxCDABE* region of pIA1250, a derivative of pIA955 with a synthetic TC_15_ cassette cloned into the leader region (9). pHK2 was transformed into selected strains with TSS (Epicentre) and plated on 20 µg/ml spectinomycin. The single colonies were inoculated into 3 ml of LB supplemented with spectinomycin and incubated at 37°C with aeration. After 8 h of growth, cultures were diluted 1:50 into EZRDM-G supplemented with 20 µg/ml spectinomycin and 0.1% arabinose and allowed to grow for 2 h. Luminescence was measured in 200-µl aliquots in triplicate on a FLUOstar Optima plate reader (BMG Labtech GmbH) and normalized by cell density. Results were analyzed with Microsoft Excel.

### β-Galactosidase assays.

Single colonies were inoculated into 3 ml of LB and incubated at 37°C with aeration overnight. Cultures were diluted 1:50 in 2 ml of EZRDM supplemented with 0.2% galactose and 1 mM isopropyl-β-d-thiogalactopyranoside (IPTG) and grown at 37°C to the mid-log phase (optical density at 600 nm [OD_600_] of ~0.5 to 0.6). The cells were pelleted and resuspended in 1 ml of Z-Buffer, 2 drops of chloroform and 1 drop of 0.1% SDS were added, and the mixture was vortexed for 10 s. Equal amounts of permeabilized cells (adjusted on the basis of cell culture density) were mixed with Z-Buffer in a 150-μl final volume in 96-well microplates (Costar). One hundred microliters of 5 mg/ml *o*-nitrophenyl-β-d-galactopyranoside was added, and β-galactosidase activity was determined from the rate of increase of the *o*-nitrophenol concentration measured every 10 s for 10 min at 420 nm in an xMark spectrophotometer (Bio-Rad). Individual cultures were assayed in triplicate, and average values of three independent cultures determined with the Microplate Manager software (Bio-Rad) are reported.
