# Supplementary material for: A Screen for rfaH Suppressors Reveals a Key Role for a Connector Region of Termination Factor Rho
Source: mBio. 2017 May 30;8(3):e00753-17. doi: 10.1128/mBio.00753-17 (PMC5449661; doi:10.1128/mBio.00753-17)
Supplement: FIG S1 [file mbo003173329sf1.pdf]

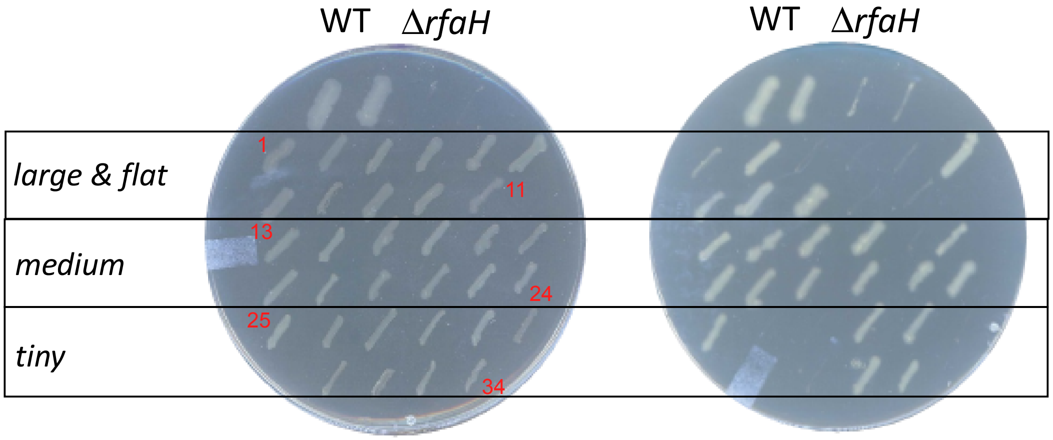

SDS, 0.25 % Novobiocin, 60 μg/ml

Novobiocin-sensitive variants

1	*rho* Δ156-158
3, 4, 5, 10	*rho* I382S
17	*rho* G150D
26	*rho* S325P
27	*rho* L285F
31, 32	*rhoL*-ΩIS2-*rho*
11	*hns* Δ25-28
