# Supplementary material for: A Screen for rfaH Suppressors Reveals a Key Role for a Connector Region of Termination Factor Rho
Source: mBio. 2017 May 30;8(3):e00753-17. doi: 10.1128/mBio.00753-17 (PMC5449661; doi:10.1128/mBio.00753-17)
Supplement: TABLE S1 [file mbo003173329st1.docx]

|  | **Mutation (nt)^1^** | **Changes in protein** | **SDS Plating Efficiency^2^** |
| --- | --- | --- | --- |
| *rfaH*^+^ |  |  | 5+ |
| *ΔrfaH* |  |  | 1+ |
| *ΔrfaH* + *yciC* | G247A | A83T | 4+ |
| *ΔrfaH* + *rpoC* | Ω[CC] at 4082 | Frameshift at residue 1361 | 5+ |
| *ΔrfaH* + *rho* | G449A | G150D; isolated twice | 5+ |
|  | G455A | G152D | 5+ |
|  | T470G | TGA at 157 | 5+ |
|  | Δ466-474 | Δ156-158; isolated thrice | 5+ |
|  | G855T | L285F | 5+ |
|  | T973C | S325P | 5+ |
|  | C1088T | S363F | 5+ |
|  | Ω[CTGCTCACGACTCAGGAAGAA] at 1128 | Ω7 at 367 | 1+ |
|  | T1145G | I382S | 4+ |
|  | ΩIS2 59 bp upstream | none | 2+ |
| *ΔrfaH* + *hns* | Δ70-81 | Δ25-28 | 4+ |
|  | T77C | L26P | 5+ |
|  | Ω[GAAACGCTGGAA] at 79 | Ω4 @27 | 5+ |
|  | Δ85-96 | Δ28-31 | 4+ |
|  | Δ103-105 | ΔV35 | 4+ |
|  | T224A | L75Q | 5+ |
|  | ΩIS1 28 bp upstream | none | 4+ |
|  | Δ*hns* | Δhns | 4+ |

**Table S1.** SDS sensitivity of Δ*rfaH* suppressors.

^1^ The position of a mutation is indicated relative to the +1 of the ORF; in the case of *hns*, the +1 corresponds to the N-terminal Met residue that is absent in the mature protein.

^2^ To assess the resistance to SDS, overnight cultures were diluted at 10^-2^, 10^-3^, 10^-4^, 10^-5^, and 10^-6^ with LB. Each diluted culture was spotted onto LB plates containing 0.003% (1), 0.016% (2), 0.08% (3), 0.4% (4), or 2% (5) SDS. The score indicates growth without apparent inhibition on the highest SDS concentration; *e.g.*, 1+ indicates efficient growth at 0.003%, and 5+ ‒ at 2% SDS.
