# Supplementary figures and images for: A Screen for rfaH Suppressors Reveals a Key Role for a Connector Region of Termination Factor Rho
Source: mBio. 2017 May 30;8(3):e00753-17. doi: 10.1128/mBio.00753-17 (PMC5449661; doi:10.1128/mBio.00753-17)

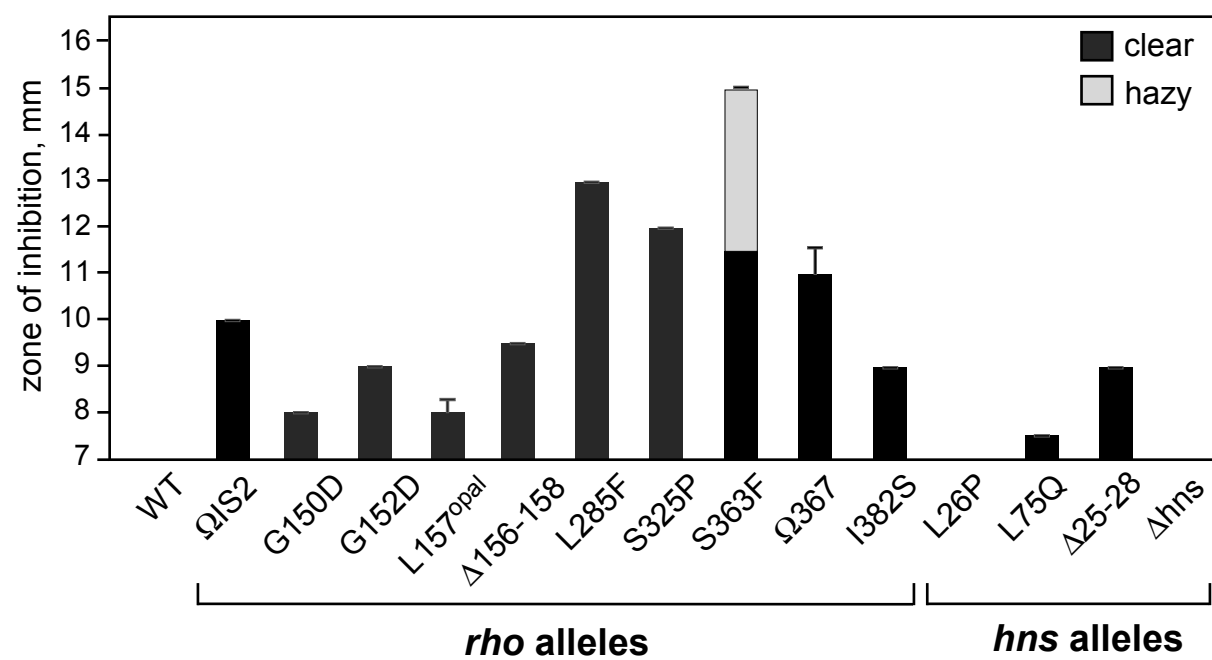

Supplement: FIG S2 [file mbo003173329sf2.pdf]

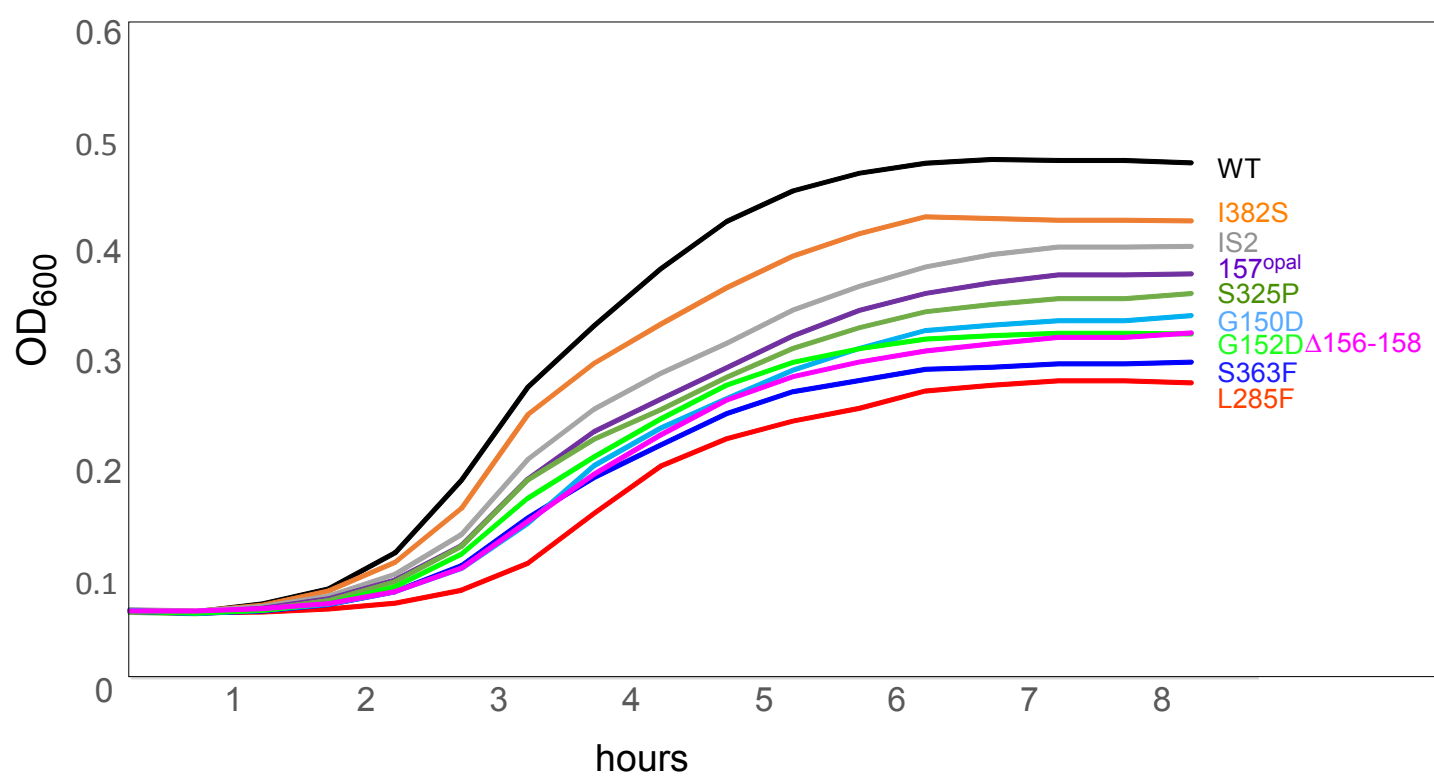

Supplement: FIG S3 [file mbo003173329sf3.pdf]

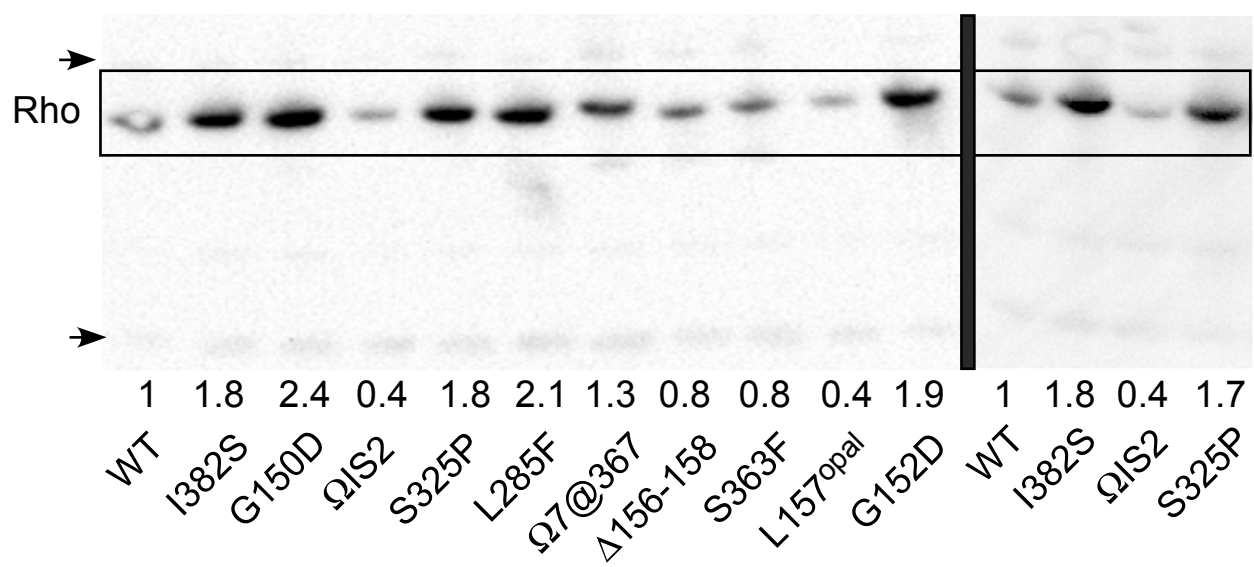

Supplement: FIG S4 [file mbo003173329sf4.pdf]

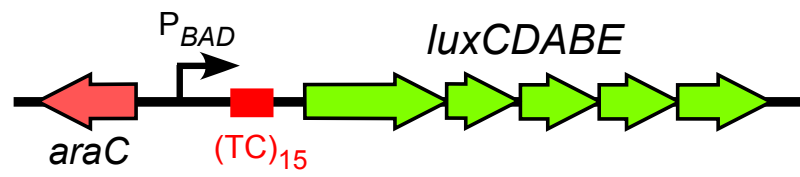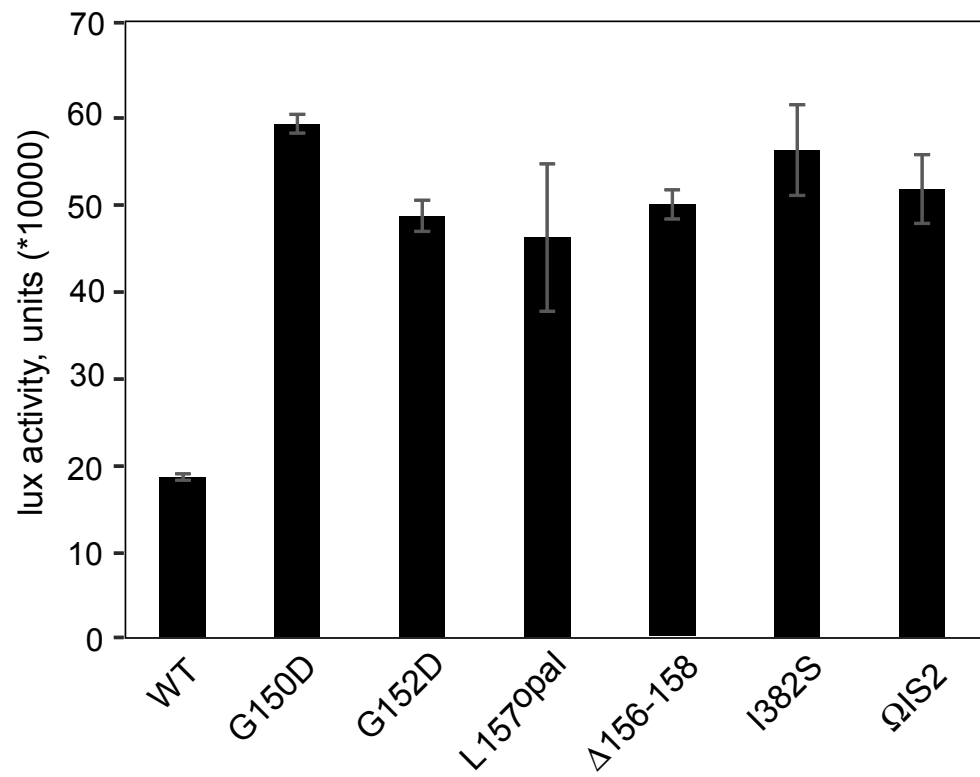

Supplement: FIG S5 [file mbo003173329sf5.pdf]

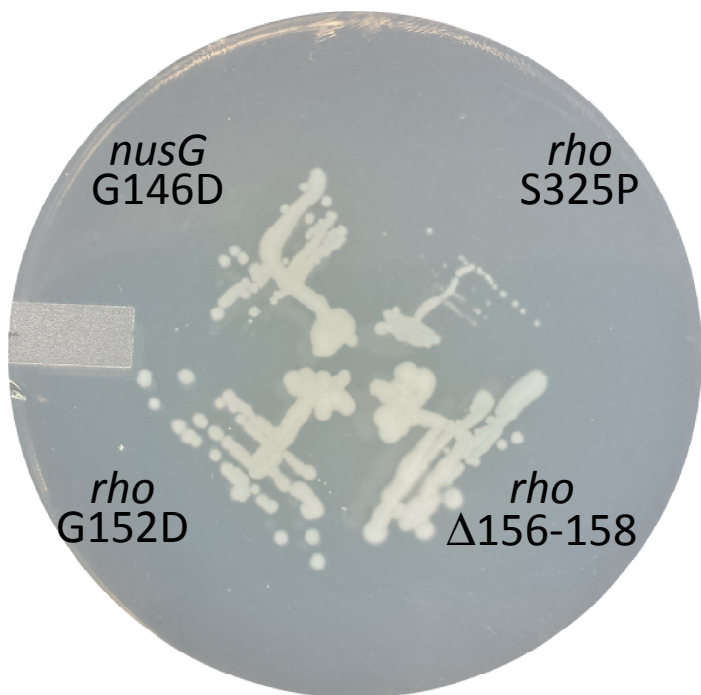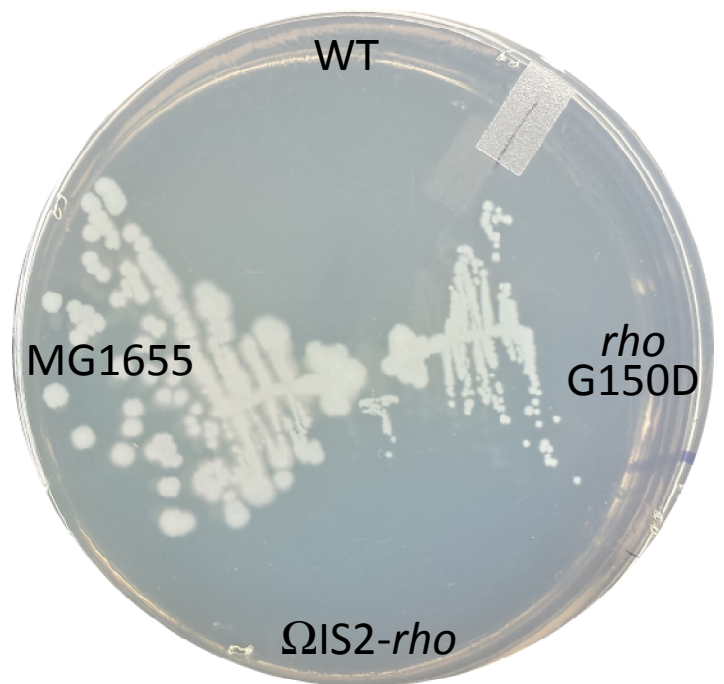

Supplement: FIG S6 [file mbo003173329sf6.pdf]
