# Supplementary material for: A Screen for rfaH Suppressors Reveals a Key Role for a Connector Region of Termination Factor Rho
Source: mBio. 2017 May 30;8(3):e00753-17. doi: 10.1128/mBio.00753-17 (PMC5449661; doi:10.1128/mBio.00753-17)
Supplement: TABLE S2 [file mbo003173329st2.docx]

**Table S2.** Strains used in this work.

| **IA strain #** | **Description** | **Source** |
| --- | --- | --- |
| 227 | MG1655 | N. Ruiz |
| 228 | MG1655 Δ*rfaH* | this work |
| 303 | MG1655 Δ*rfaH* *rho* I382S | this work |
| 304 | MG1655 Δ*rfaH* *hns* ΔV35 | this work |
| 305 | MG1655 Δ*rfaH* *rho* G150D | this work |
| 306 | MG1655 Δ*rfaH* *rhoL*-ΩIS2-*rho* | this work |
| 307 | MG1655 Δ*rfaH* *yciC* T83S | this work |
| 329 | MG1655 Δ*rfaH* *hns* ΩETLE@27 | this work |
| 330 | MG1655 Δ*rfaH* *hns* Δ25-28 | this work |
| 331 | MG1655 Δ*rfaH* *rpoC* 1361-frameshift | this work |
| 333 | MG1655 Δ*rfaH* *rho* S325P | this work |
| 334 | MG1655 Δ*rfaH* *rho* L285F | this work |
| 336 | MG1655 Δ*rfaH* *hns*::Kan | this work |
| 338 | MG1655 Δ*rfaH* *rho* ΩEELLTTQ@367 | this work |
| 340 | MG1655 Δ*rfaH* *rho* Δ156-158 | this work |
| 341 | MG1655 Δ*rfaH* *rho* S363F | this work |
| 342 | MG1655 Δ*rfaH* *rho* L157^opal^ | this work |
| 344 | MG1655 Δ*rfaH* *rho* G152D | this work |
| 349 | MG1655 Δ*rfaH* *hns* L26P | this work |
| 350 | MG1655 Δ*rfaH* *hns* L75Q | this work |
| 351 | MG1655 Δ*rfaH* *hns* Δ25-28 | this work |
| 352 | MG1655 Δ*rfaH* ΩIS1-*hns* | this work |
| 381 | W3110 *gal490** *nusG* G146D *rpoC*::Kan | M. Gottesman |
| 383 | MG1655 *nusG* G146D *rpoC*::Kan | this work |
| 384 | MG1655 Δ*rfaH* *nusG* G146D *rpoC*::Kan | this work |
| 392 | MC4100 *galEp3* λRS45: P*_lac_*-H19B-T_R1_-*lacZYA* (JG5147) | J. Gowrishankar |
| 393 | MC4100 *galEp3* *nusG* G146D λRS45: P*_lac_*-H19B-T_R1_-*lacZYA* (JG5153) | J. Gowrishankar |
| 404 | MC4100 *galEp3* λRS45: P*_lac_*-H19B-T_R1_-*lacZYA rho* I382S *ilvC*::Kan | this work |
| 405 | MC4100 *galEp3* λRS45: P*_lac_*-H19B-T_R1_-*lacZYA* *rho* G150D *ilvC*::Kan | this work |
| 406 | MC4100 *galEp3* λRS45: P*_lac_*-H19B-T_R1_-*lacZYA* *rhoL*-ΩIS2-*rho* *ilvC*::Kan | this work |
| 413 | MC4100 *galEp3* λRS45: P*_lac_*-H19B-T_R1_-*lacZYA* *rho* L157^opal^ *ilvC*::Kan | this work |
| 414 | MC4100 *galEp3* λRS45: P*_lac_*-H19B-T_R1_-*lacZYA rho* G152D *ilvC*::Kan | this work |
| 415 | MC4100 *galEp3* λRS45: P*_lac_*-H19B-T_R1_-*lacZYA* *rho* Δ156-158 *ilvC*::Kan | this work |
| 418 | MC4100 *galEp3* λRS45: P*_lac_*-H19B-T_R1_-*lacZYA* *ilvC*::Kan | this work |
| 431 | MG1655 Δ*rfaH* *rhoL*-ΩIS2-*rho* *ilvC*::Kan | this work |
| 432 | MG1655 Δ*rfaH* *rho* G150D *ilvC*::Kan | this work |
| 433 | MG1655 Δ*rfaH* *rho* G152D *ilvC*::Kan | this work |
| 434 | MG1655 Δ*rfaH* *rho* L157^opal^ *ilvC*::Kan | this work |
| 435 | MG1655 Δ*rfaH* *rho* Δ156-158 *ilvC*::Kan | this work |
| 439 | MG1655 Δ*rfaH* *rho* I382S *ilvC*::Kan | this work |
| 440 | MG1655 Δ*rfaH* *ilvC*::Kan | this work |

1. IA228 was constructed from IA227 by P1 transduction of the *rfaH*::Kan allele from the Keio collection and subsequent flipping-out of the kanamycin-resistant cassette.
2. Strains IA303-352 are original isolates of spontaneous SDS-resistant (SDS^R^) suppressors of IA228.
3. Strains 383 and 384 were constructed by P1 transduction of *rpoC*::Kan closely linked to *nusG* G146D allele from IA381 into IA227 and 228. The transductants were sequenced to confirm the presence of the G146D substitution.
4. Strains IA404-418 were constructed by P1 transduction of *ilvC*::Kan marker from IA228 and its SDS-resistant derivatives into JG5147 (IA392). The *lac*^+^ transductants were sequenced to confirm the presence of *rho* mutations.
5. Strains IA431-440 were constructed by P1 transduction of *ilvC*::Kan marker from IA228 and its SDS^R^ derivatives into IA228 (SDS^S^). The SDS^R^ transductants were sequenced to confirm the presence of *rho* mutations.
